# On and Off switches in the brain

**DOI:** 10.3389/fnbeh.2015.00114

**Published:** 2015-04-29

**Authors:** Ahmed A. Moustafa

**Affiliations:** ^1^Department of Veterans AffairsEast Organge, New Jersey, USA; ^2^Marcs Institute for Brain and Behaviour and School of Social Sciences and Psychology, University of Western SydneySydney, NSW, Australia

**Keywords:** basal ganglia, motor processes, sleep, fear, cognition

The brain seems to have evolved one class of neurons that initiate certain behaviors, and another class of neurons that do the opposite (i.e., inhibit these behaviors). In other words, our brains seem to be equipped with different On and Off switch neurons. These neurons are found across many domains, including motor, cognitive, emotional, sleep, and others. This is contrasted to evolving one type of neurons that can do both functions (e.g., motor neurons that can initiate movement where active and inhibit movement when inactive). First, I review neuroscience experiments reporting On and Off switch neurons. Second, I discuss why this is a good design for implementing many behavioral processes than just relying on one kind of neurons that regulate opposing behaviors.

## Motor control

As an example, human studies have shown that there are two types of neurons in the striatum (input structure of the basal ganglia) that play a role in either stimulating or inhibiting motor processes, often known as On and Off motor neurons or Go vs. NoGo neurons (Albin et al., 1989; Frank, 2005; Frank et al., 2007a,b; Moustafa et al., 2008a; Cox et al., 2015). These are suggested to correspond to dopamine D1 and D2 neurons in the striatum, respectively (Frank et al., 2007a). These findings are also supported by animal studies showing On and Off motor neurons in the striatum (Shen et al., 2008; Kravitz et al., 2010; Lobo et al., 2010). Interestingly, various studies also show that the cortex has two different regions for initiating or inhibiting motor responses, which are respectively the motor cortex vs. lateral prefrontal cortex (Sakagami et al., 2006; Aron, 2007). Single-cell recording studies suggest the lateral prefrontal cortical regions have off motor switches (Sakagami et al., 2001).

## Fear responses

Findings similar to the On and Off motor switch neurons were also reported for other behavioral domains. For example, the amygdala was found to have two classes of neurons that either initiate or inhibit fear responses (Pare et al., 2004; Anglada-Figueroa and Quirk, 2005; Herry et al., 2008; Amano et al., 2010; Moustafa et al., 2013a). For example, Pare and colleagues reported a class of neurons, known as the intercalated (ITC) cells, that were found to inhibit fear responses (Pare and Smith, 1993; Amir et al., 2011), although recent studies found conflicting results (Strobel et al., 2015)This is contrasted with other kinds of neurons in the basolateral amygdala that play a role in initiating fear responses (Maren et al., 1996; Sierra-Mercado et al., 2010). The differences among these neural populations is that the intercalated neurons send inhibitory, while the fear-expression basolateral neurons, send excitatory, projections to the central nucleus of the amygdala, which initiate fear responses (e.g., regulate changes in heart rate responses, breathing, skin conductance). Importantly, some studies also report that the central nucleus of the amygdala may have On and Off neurons (Haubensak et al., 2010). Interestingly, the same dissociation for amygdala neurons responsible for initiating and inhibiting fear responses were also reported for cortical structures, including respectively, the prelimbic and infralimbic cortices (Vidal-Gonzalez et al., 2006; Laurent and Westbrook, 2009; Sierra-Mercado et al., 2010), which respectively project to fear-expression and intercalated neurons in the BLA(Berretta et al., 2005). Thus, the brain seems to have evolved On and Off fear neurons that help us adapt in different environments.

Importantly, posttraumatic stress disorder is related to deficiency to extinguish and inhibit fear responses, which is controlled by projections from the infralimbic cortex to intercalated neurons and/or projections from intercalated neurons to the central nucleus of the amygdala (Norrholm et al., 2011). Like posttraumatic stress disorder, there are studies that suggest that addiction is also related to an impairment in extinguishing drug-seeking behavior (as analogous to non-extinguished fear memories in posttraumatic stress disorder) (Peters et al., 2009). So it is possible that an impairment in the Off switches in the amygdala and nucleus accumbens may respectively underlie some of the symptoms in posttraumatic stress disorder and addiction. It remains to be shown which neural populations play a role in drug-seeking vs. inhibition of drug-seeking behavior.

## Sleep

Interestingly, it has also been reported that the brain has On and Off neurons in the lateral hypothalamus that regulate sleep (Hassani et al., 2009). These are known as melanin-containing and orexin neurons. It was found that the melanin-containing neurons are more active during sleep states (particularly during slow wave sleep) while orexin neurons are active during wake state (Adamantidis et al., 2007), that is, these represent On and Off sleep neurons. These findings are supported by other studies showing that orexin overexpression is related to insomnia (Prober et al., 2006) and that orexin deficiency is related to narcolepsy (Chemelli et al., 1999). Interestingly, orexin antagonists are being trialed for the treatment of insomnia (Cox et al., 2010; Winrow et al., 2012; Winrow and Renger, 2014). Further, the function of these neurons may help regulate other brain regions (e.g., inhibit or stimulate motor areas) during wake vs. sleep states. Importantly, other studies found that other neurotransmitters do also play a role in the initiation of wake vs. sleep states. For example, work by Foster and colleagues show that galanine and GABA can also act as sleep switch, as both are suppressed during sleep (Wulff et al., 2010). The same authors also found that adenosine plays a role in switching from wake to sleep states. It remains to be shown whether adenosine impacts orexin and melanin-containing neurons, or vice versa during sleep, and whether these various neurochemicals play dissociable roles during sleep.

## Memory and cognition

In terms of cognitive processes, the findings are less clear, but there are some indication that the brain could have evolved On and Off neurons to stimulate or inhibit certain kinds of cognitive processes. In the working memory domain, for example, it was suggested by theoretical analyses and experimental data that some neurons in the striatum play a role in gating information into working memory, while others inhibit information from being maintained in working memory (Frank et al., 2001; Frank and O'Reilly, 2006; Moustafa et al., 2008b). The working memory inhibition mechanism is assumed to play a key role in minimizing distractibility, and may explain cognitive deficits and the occurrence of positive symptoms (hallucinations and misperception) in schizophrenia, yet, to my knowledge, very few studies have attempted to study its neural substrates. The same working memory gating and inhibition mechanism has been also suggested for attentional processes, that is, dopamine D1 receptor neurons aid in paying attention to stimuli in the environment but dopamine D2 receptor neurons inhibit paying attention (Moustafa et al., 2008b). To the best of my knowledge, I do not know whether this has been reported in experimental studies.

As for long-term memory, although most studies focus on understanding the neural mechanism of memory retrieval, few studies have investigated memory retrieval vs. memory suppression, that is, to understand the mechanism of turning the switch on to retrieve memory or turning it off to suppress memory retrieval (Anderson and Green, 2001; Levy and Anderson, 2008; Benoit and Anderson, 2012). The importance of turning off (i.e., suppressing) memory retrieval is linked to trauma-related disorders, such as posttraumatic stress disorder, when it is potentially important not to remember negative life events. It has been suggested that different parts of the prefrontal cortex play a role in memory retrieval and memory suppression (Depue et al., 2007). A recent study (Benoit and Anderson, 2012) investigated the neural mechanisms of memory suppression vs. thought substitution (i.e., a controlled retrieval mechanism where subjects recall one event in order to avoid recalling another event, which is arguably a form of memory retrieval). Benoit and Anderson (2012) suggest that two different prefrontal mechanisms may be responsible for these processes. Although, these brain imaging studies suggest there are two neural mechanisms for memory retrieval and suppression, to my knowledge, we do not know whether there are two types of neurons that switch On and Off memory retrieval. The previously-mentioned brain imaging studies on memory found that dorsolateral prefrontal cortex plays a role in both memory recall and suppression. It is not known whether there are different neurons in this brain area that regulate these processes. As most, if not all, studies on memory recall and suppression were conducted in humans, our knowledge on its neural substrates are limited. Future optogenetic studies can study whether there are different dorsolateral prefrontal cortex neurons that play a role in memory recall vs. suppression. Further, as in fear and motor responses, it is possible these neurons are intermingled in the dorsolateral prefrontal cortex.

## Rewarding vs. aversive stimuli processing

The story is a bit more complex for affective processes, such as responding to rewarding vs. aversive stimuli. Although some studies found different neurons respond to rewarding vs. aversive stimuli (Frank et al., 2007a; Hikida et al., 2010; Kravitz et al., 2012; Cox et al., 2015), other studies found that one class of neurons can represent rewarding vs. aversive information across one dimension (Tom et al., 2007; Morrison and Salzman, 2009). The rewarding vs. aversive neurons may play a role in activating/inhibiting switches, such as on vs. off fear responses, or Go vs. NoGo motor plans. For example, studies suggest that orbitofrontal neurons representing rewarding vs. aversive information may project to Go vs. NoGo motor neurons in the striatum (Frank and Claus, 2006), although this assumption should be tested experimentally.

## Perspective

Across all of the behavioral domains mentioned above, the On and Off switches are intermingled in the same brain region, including dopamine D1 and D2 neurons in the striatum, intercalated and fear-expression basolateral amygdala neurons, as well as orexin and melanin-containing neurons. Table 1 summarizes these data along with associated pathologies.

**Table 1:** **On and off neurons and associated brain disorders**.

**Behavioral domain**	**Brain regions (On and Off switches)**	**Related brain disorders**
Fear	Amygdala (fear-expression and intercalated neurons)	Posttraumatic stress disorder
Motor control	Striatum (dopamine D1 and D2 neurons)	Parkinson's disease and schizophrenia.
Working memory	Striatum (dopamine D1 and D2 neurons)	–
Episodic memory	Dorsolateral prefrontal cortex	–
Sleep	Lateral hypothalamus (melanin-containing and orexin neurons)	Insomnia, narcolepsy

What are the implications of these findings? It is important to know that some clinical disorders impact the Off switches, as described above. One example is Parkinson's disease, where the Off switch (i.e., the basal ganglia indirect pathway) is active and thus movement is hard to initiate (Albin et al., 1995). As another example, some studies found that schizophrenia is associated with impaired D2 receptors (Seeman and Kapur, 2000; Silvestri et al., 2000) suggesting that the Off switch is not working properly, and thus no limit is put on attentional or perceptual processes, potentially causing hallucinations and misperception. However, there are debates regarding whether these findings are related to schizophrenia or the administration of antipsychotics (Abi-Dargham et al., 2000; Seeman and Kapur, 2000). Similarly, it was found that posttraumatic stress disorder is associated with dysfunctional dopamine D2 receptors (i.e., impaired Off switches), possibly explaining the occurrence of intrusive thoughts in this disorder (Comings et al., 1996; Lawford et al., 2006). This is corroborated by studies showing that antipsychotics (which work on dopamine D2 receptors) were found to minimize posttraumatic stress disorder symptoms (Ahearn et al., 2003; Adetunji et al., 2005), thus possibly “fixing” Off switches. Given that psychopharmacological agents target D2 receptors (with varying affinity to D1 receptors) and that D2 neurons we found to play a role in inhibition processes, it is important to understand the function of “off switches” in the brain, as this may aid in also understanding and treating various neuropsychological disorders (for discussion see Moustafa et al., 2013b).

These findings of On and Off switch neurons can have implications for building intelligent machines. Most existing models of motor control, for example, focus on the simulation of the initiation of motor responses, but often do not incorporate mechanisms of motor inhibition (Gupta and Noelle, 2007). The design of more complex motor systems may in the future require the integration of On and Off motor switches, with segregated inputs and outputs to each one, that can possibly show human-like motor control behavior.

As reported above, the On and Off switches were found in cortical and subcortical structures even in one behavior domain, such as motor control, affective processes, and fear responses. It is not known why the brain could have evolved at least two sets of On and Off switches for the same kind of processes! It is possible that these switches are controlled by different inputs and regulate different outputs. However, in the fear response domain, it was suggested that the prelimbic and infralimbic (cortical On and Off switches) structures respectively control the basolateral and intercalated cells, that is On and Off switches (Pare et al., 2004; Moustafa et al., 2013a). Another potential explanation here is that the brain has a hierarchical structure with cortical areas possibly controlling subcortical switches. However, more research is needed to study the relationship among neurons in both cortical and subcortical structures, and how they are impacted by environmental inputs.

Now, it is not known why the brain did not evolve only one type of neurons that control On and Off switches, akin to light switches, for turning light On and Off. Importantly, it is more computationally intensive to use two types of switches rather than one. The potential value of having two classes of On and Off switches in the brain is possibly for better control of afferent and efferent projections. For example, with one class of neurons regulating wakefulness vs. sleep, it is hard to send information to efferent systems to regulate their activities (e.g., if it is wake state, activate motor and cognitive areas. If it is a sleep state, inhibit motor areas and activate hippocampus for memory consolidation). The same logic applies to inputs coming into On and Off switches. In order to segregate the kinds of inputs that turn On and Off the switches, having them controlled by separate neurons is most likely a better design. Although it is possible to design a system with one type of neurons that control On and Off functions, this control mechanism will be more difficult to adjust and the potential for error is large. Confusing Go with NoGo actions, for example, can be deadly, if one attempts to run away from predator, but the right key is not turned on.

It is important to note that the On and Off switches are only a small part of the neural mechanism underlying the processes described here. Each behavioral domain involves additional complex processes besides the On and Off switches. For example, in the motor domain, there are brain regions that play a role in motor preparation and execution. These regions eventually impact the functioning of the On and Off switches. It is also important to note that the existence of On and Off switches in the brain does not imply a binary response. There are probably graded responses within both initation vs. inhibition responses. For example, a snake 4 m away from us may initiate a weaker fear response than a snake 2 m away. Similar graded responses can be found in the motor and memory systems across on and off switches.

It is possible that the brain has evolved On and Off switches, as there are evidence such neurons do exist in fish and birds (and not only in rats and humans). For example, striatal D1(Go) and D2(NoGo) neurons are also found in fish (Ericsson et al., 2013) birds (Ding and Perkel, 2002), and turtles (Barral et al., 2010). As for fear, one study has reported intercalated cells in chicken with similar anatomical structures to those of mammals, suggesting perhaps they play a similar function across species (Vicario et al., 2014). Still, however, more research is needed to identify these neurons in other animals as well as across other behavioral domains.

### Conflict of interest statement

The author declares that the research was conducted in the absence of any commercial or financial relationships that could be construed as a potential conflict of interest.
